# Biomarkers in Cervical Cancer

**Published:** 2007-02-07

**Authors:** Eun-Kyoung Yim, Jong-Sup Park

**Affiliations:** Department of Obstetrics and Gynecology, Catholic University Medical College, 505 Banpodong, Seochogu, Seoul, 137-040, Republic of Korea

**Keywords:** Cervical cancer, Human papillomavirus (HPV), biomarker

## Abstract

Cervical cancer, a potentially preventable disease, remains the second most common malignancy in women worldwide. Human papillomavirus (HPV) is the single most important etiological agent in cervical cancer, contributing to neoplastic progression through the action of viral oncoproteins, mainly E6 and E7. Cervical screening programs using Pap smear testing have dramatically improved cervical cancer incidence and reduced deaths, but cervical cancer still remains a global health burden. The biomarker discovery for accurate detection and diagnosis of cervical carcinoma and its malignant precursors (collectively referred to as high-grade cervical disease) represents one of the current challenges in clinical medicine and cytopathology.

## Introduction

Worldwide, cervical cancer is the second most common cancer in women; and is estimated to cause over 470,000 new cases and 233,000 deaths each year. Based on strong epidemiological evidence, supported by basic experimental findings, there is no doubt that persistent infections with high-risk types of human papillomavirus (HPV) represent a necessary cause of cervical cancer (Walboomers et al. 1999). HPVs infect epithelial cells and cause a variety of lesions ranging from common warts to cervical neoplasia and cancer. Over 100 different HPV types have been identified so far, with a subset of these being classified as high risk. High-risk HPV DNA is found in almost all cervical cancers (>99.7%), with HPV16 being the most prevalent type in both low-grade disease and cervical neoplasia. Productive infection by high-risk HPV types is manifest as cervical flat warts or condyloma that shed infectious virions from their surface. Viral genomes are maintained as episomes in the basal layer, with viral gene expression being tightly controlled as the infected cells move towards the epithelial surface. The pattern of viral gene expression in low-grade cervical lesions resembles that seen in productive warts caused by other HPV types. High-grade neoplasia represents an abortive infection in which viral gene expression becomes deregulated, and the normal life cycle of the virus cannot be completed. Most cervical cancers arise within the cervical transformation zone at the squamous/columnar junction, and it has been suggested that this is a site where productive infection may be inefficiently supported (Doorbar, 2006).

Although HPV infection is widespread, few people even know they are infected as the symptoms are seldom noticeable. It is even less well known is that nearly all cervical cancers (99.7%) are directly linked to previous infection with one or more of the oncogenic types of HPV (Walboomers et al. 1999). It is estimated that for every 1 million women infected, a hundred thousand (about 10%) will develop precancerous changes in their cervical tissue. Of these, about 8% of them will develop early carcinoma limited to cervical epithelium (carcinoma *in situ*; CIS) and a few of them will develop invasive cancer unless the precancerous lesions are detected and treated with such cases having been found to carry the oncogenic HPVs (e.g. types 16 and 18) that cause cervical cancer.

The HPV genome consists of 8 kb, and is a double-stranded DNA molecule. The relative arrangement of the 8–10 open reading frames (ORFs) within the genome is the same in all papillomavirus types, and a particular characteristic of papilloma viruses is that the partly overlapping ORFs are arranged on only one DNA strand. The genome can be divided into three regions: the long control region (LCR) without coding potential; the region of early proteins (E1–E8); and the region of late proteins (L1 and L2) (Walter and Philip, 2004). E6 and E7 are the most important oncogenic proteins. These proteins have pleiotropic functions, such as transmembrane signaling, regulation of the cell cycle, transformation of established cell lines, immortalization of primary cell line and regulation of chromosomal stability. Both E6 and E7 proteins can bind to multiple cellular targets. The interactions that are thought to be most relevant for their transforming functions are E6 binding, via the cellular protein E6-AP, to the tumor suppressor gene product p53, and E7 binding to the retinoblastoma tumor suppressor gene product pRb and its related pocket proteins, p107 and p130 (Dyson et al. 1989; Davies, 1993). The first interaction results in rapid ubiquitin-dependent proteolytic degradation of p53, which prevents cells from undergoing p53-mediated apoptosis (Thomas, 1999). A consequence of E7-pRb interaction is interfering with cell cycle control. In combination, the E6-p53 and E7-pRb interactions seem to compromise the accuracy of mitosis. In addition, HPV E6 can activate the telomere lengthening enzyme telomerase independent of p53 binding, and E7 can induce abnormal centrosome duplication through a mechanism independent of inactivation of pRb and its family member. It is likely that these latter properties also contribute to the transforming characteristic of these viral oncoproteins.

HPV infection causes changes in expression of host cervical cell cycle regulatory proteins. Such differentially expressed host proteins and nucleic acids may have a role as ‘biomarker’ of dysplastic cells. Investigation of potential biomarkers may also help to unravel new pathways involved in the HPV-mediated pathogenesis of cervical dyskaryosis.

## Cervical Cancer Screening

For more than 50 years the Pap smear has been the mainstay of cervical screening resulting in a dramatic decrease in death from cervical cancer. However, the Pap smear has certain disadvantages (Table 1). It has a low sensitivity and high false negative rate. The data reveals that some of the false negative Pap smears rarely contain any abnormal cells on the slide (DeMay, 1996; Spitzer, 2002). So far, an effort to seek an explanation for this matter has been focused on either the incomplete transfer of cells from collection devices to the slide or inadequate sampling. This results in the development of liquid-based cytology technique (DeMay, 1996; McGoogan et al. 1998). Additionally, one of the emerging explanations is the lack of exfoliation of dysplastic cell (shedder and nonshedder hypothesis) (Felix et al. 2002; Felix, 2003). Data from some studies have been shown to the effect that there is an abnormal expression of the adhesion molecules in a subset of dysplastic lesions of the cervix (et al. 2002; Felix, 2003). It can prevent detection by any test requiring exfoliated abnormal cell, including liquid-based technique. Despite the nonshedding behavior, those lesions can be identified by visual test (Felix, 2003). There have been a number of visual tests which investigated for primary screening or used as adjunctive test of cytology method. These tests include cervicography, visual inspection with acetic acid (VIA), speculoscopy. At the present time, cervicography has a limited role as a primary screening or an adjunct to Pap smear (Schneider et al. 1999; Autier et al. 1999; Costa et al. 2000). However, as a triaging strategy for patients with ASCUS Pap smear, it is still a promising technique (Ferris et al. 2001; Brotzman, 2002). Direct inspection is the other method based on applying acetic acid to the cervix and then visualizing it. It can be done under incandescent light with or without magnification or the chemiluminescent light (Wright et al. 2002; Parham, 2003). This chemiluminescent light is of low intensity. It is diffuse and produces minimal reflective glare from normal tissue. There are studies showing that the use of chemiluminescent light allows the examiner to identify acetowhitening better than the incandescent light does (Lonky and Edwards, 1992; Mann et al. 1993; Lonky, 2002; Parham, 2003). Speculoscopy is developed for cervical screening by using chemiluminescence and low-power magnification to examine the cervix after applying an acetic acid. It can detect acetowhite dysplastic lesions and has been reported to be effective in detecting cervical intraepithelial lesions when combined with the Pap smear (Lonky, 2002; Wright et al. 2002; Parham, 2003).

Presently, new technologies such as liquid-based cytology, HPV DNA test have been introduced. This test is used to detect the HPVs, which is considered the primary cause of virtually all cervical cancers. There are at least 30 different types of HPV strains that target the genital area, and are transmitted through sexual, skin-to-skin contact. Of these, approximately 13 are considered to be ‘high risk’ because they can trigger the development of abnormal cells associated with cervical cancer. The remaining ‘low-risk’ types can cause genital warts. Although the Pap smear can pick up the cellular changes caused by high-risk types of HPV, it’s not as sensitive as the HPV test, which specifically detects the viral DNA. The HPV test is not yet routinely used by the majority of doctors, in part because it is more expensive than a regular Pap test. Therefore, it would be important to improve the cost-effectiveness of screening and reduce the psychologic burden of benign positive test results.

## Molecular Biomarkers in Cervical Cancer

### HPV E6

**Figure 1:**
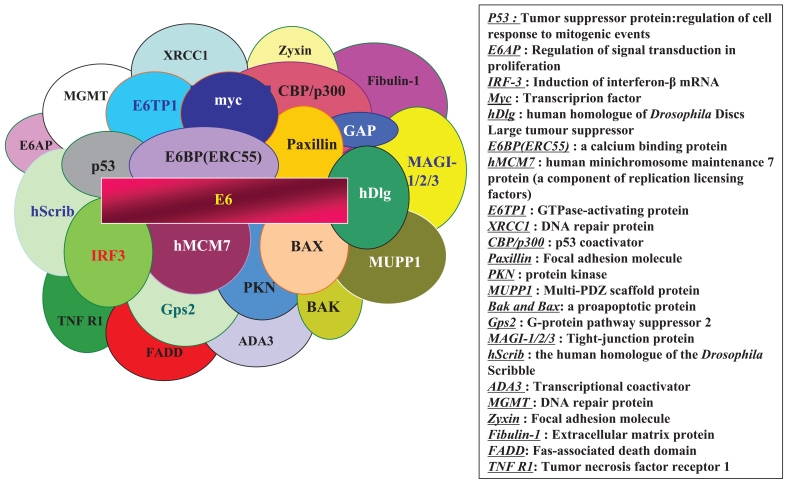
Cellular binding partners for HPV E6.

The E6 oncoproteins of high risk HPV interfere with the function of the cellular tumor suppressor protein p53 through the induction of increased proteasome-dependent p53 degradation. High risk HPV E6 proteins target the cellular E3 ubiquitin ligase E6-AP to p53, resulting in the transfer of ubiquitin peptides from E6-AP to p53, which marks p53 for degradation by the 26S proteasome. Low risk and cutaneous epithelia-infecting HPV E6 proteins are unable to target the cellular p53 protein for degradation through the proteasome pathway. Although E6-induced loss of p53 is an important element of E6-induced cellular transformation, recent studies have identified a number of additional cellular targets of E6 that may also play an important role. These included the following (Filippova et al. 2004; Yim et al. 2004): proteins involved in the regulation of transcription and DNA replication, such as p300/CBP (Huang and McCance, 2002), Gps2 (Degenhardt and Silverstein, 2001), IRF-3 (Ronco et al. 1998), hMcm7 (Kukimoto et al. 1998), E6TP1 (Gao et al. 1999) and ADA3 (Kumar et al. 1999); proteins, involved in apoptosis and immune evasion, such as Bak (Thomas and Banks, 1998), Bax (Bemard et al. 2003), TNF receptor 1 (TNF R1), FADD (Filippova et al. 2002) and c-Myc (Chen and Defendi, 1992); proteins involved with epithelial organization and differentiation, such as paxillin (Tong and Howley, 1997), E6BP/ERC-55 (Chen et al. 1995), zyxin (Degenhardt and Silverstein, 2001) and fibulin-1 (Du et al. 2002); proteins involved in cell-cell adhesion, polarity and proliferation control, which contain a PDZ-binding motif, such as hDLG (Kiyono et al. 1997), hScrib (Nakagawa and Huibregtse, 2000), PKN (Gao et al. 2000), MAGI-1 (Glaunsinger et al. 2000), MAGI-2, MAGI-3 (Thomas et al. 2002) or MUPP1 (Lee et al. 2000); and proteins involved in DNA repair, such as XRCC1 (Iftner et al. 2002) and 6-*O*-methylguanine-DNA methyltransferase (MGMT) (Strivenugopal and Ali-Osman, 2002) (Figure 1).

### HPV E7

**Figure 2:**
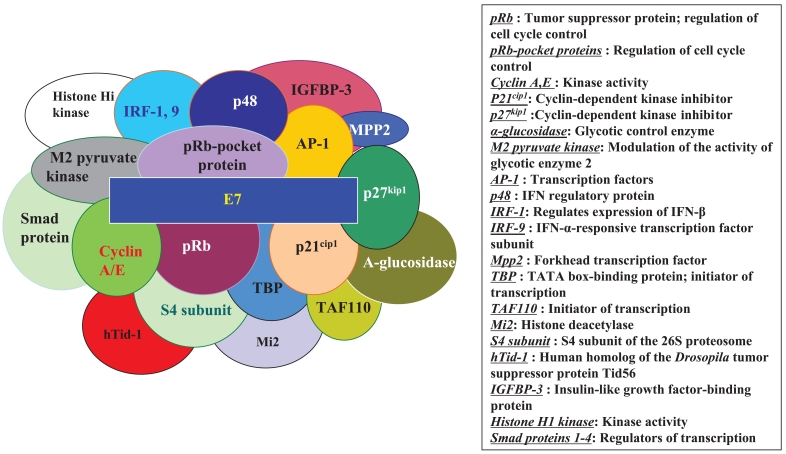
Cellular binding partners for HPV E7.

HPV E7 proteins interact with the so-called ‘pRb-associated pocket proteins,’ including the retinoblastoma protein pRb, which are negative cell cycle regulators involved in the G1/S and G2/M transitions. The interaction between high-risk E7 and pRb results in enhanced phosphorylation and degradation. pRb destruction leads to the release of E2F family of transcription factors and the subsequent activation of genes promoting cell proliferation. However, the stimulatory effect of E7 upon cell proliferation not only depends on its association with pRb, since E7 targets the function of a plethora of cell cycle regulators, including cyclin A (Dyson et al. 1992), E (McIntyre et al. 1996) and cyclin-dependent kinase inhibitor p21^Cip1^ (Jones et al. 1997) and p27^kip1^ (Zerfass-Thome et al. 1996) together with the metabolic regulators, acid-α-glucosidase (Zwerschke et al. 2000) and M2 pyruvate kinase (Zwerschke et al. 1999). HPV E7 also interferes with the activity of a variety of cellular transcription factors, such as AP-1 (Antinore et al. 1996), p48 (Bamard and McMillan, 1999), interferon regulatory factor-1 (IRF-1) (Part et al. 2000), forkhead transcription factor MPP2 (Luscher-Firzlaff et al. 1999), TATA- box binding protein (TBP) and TATA-box binding protein- associated factor (TAF110) (Mazzarelli et al. 1995), as well as with the Mi2 histone deacetylase (Brehm et al. 1999). Also, E7 interacts with the S4 subunit of the 26S proteasome (Duensing and Munger, 2003), a human homolog of the *Drosophila* tumor suppressor protein Tid56 (hTid-1) (Schilling et al. 1998), interferon regulatory factor-9 (IRF-9) (Antonsson et al. 2006), Smad protein (Habig et al. 2006), insulin-like growth factor binding protein (IGFBP-3) (Mannhardt et al. 2000) and histone H1 kinase (Davies et al. 1993) (Figure 2).

### Mini chromosome maintenance (MCM)

DNA replication occurs only once in a single normal cell cycle, due to a mechanism known as ‘licensing’ of DNA replication. This process requires the assembly of a protein complex which includes the mini chromosome maintenance (MCM) proteins and the cell division cycle protein 6 (CDC6) (Cook et al. 2002; Shin et al. 2003). Disassembly of this complex prevents repetitive replication during the same cell cycle (Lei and Tye, 2001). Changes in the expression pattern of DNA ‘licensing’ proteins are frequently observed in dysplastic cells. In comparison with be present only during the cell cycle in normal cells, MCM proteins and CDC6 have been demonstrated to be overexpressed in dysplastic cells.

In normal cervical epithelium, MCM protein staining is limited to the basal proliferating layer and is absent in differentiated and quiescent cells. In cervical glandular and squamous dysplasia, however, MCM expression is dramatically increased, suggesting its potential as a biomarker of cervical dysplasia (Ohta et al. 2001; Stoeber et al. 2002; Going et al. 2002; Alison et al. 2002; Davies et al. 2002; Davidson et al. 2003). MCM5 has been the focus of much of this research, but MCM7 is also a highly informative marker of cervical cancer. The number of nuclei positive for MCM5 at the surface of dysplastic epithelium correlates with the severity of dysplasia (Williams et al. 1998; Freeman et al. 1999) (Table 2).

### Cell division cycle protein 6 (CDC6)

Both MCM5 and CDC6 play essential roles in the regulation of eukaryotic DNA replication. CDC6 was first identified in 1998 as a marker of cervical dysplastic cells in cervical biopsies and in smears using polyclonal antibodies. Not only MCM5 but also CDC6 protein expression are present in proliferating cells and absent in differentiated or quiescent cells. In normal cervical epithelium, CDC6 staining is absent or limited to the basal proliferative layer. However, CDC6 protein expression is dramatically up-regulated in squamous and glandular cervical carcinomas. Several studies have illustrated a linear increase in CDC6 expression observed in normal cervix, preinvasive neoplasia and invasive cervical carcinoma. CDC6 was preferentially expressed in areas exhibiting histological HPV changes. Interestingly, the expression pattern of CDC6 closely mirrors that of the high-risk HPV E6 oncoprotein, which is mainly expressed in higher grade lesions and invasive carcinomas (Table 3).

### p16^INK4A^

p16^INK4A^ is a tumor supressor gene and a key regulator of the cell cycle. The expression pattern of p16^INK4A^ in dysplastic squamous and glandular cervical cells in tissue sections and in cervical smears has been extensively investigated (Sano et al. 1998; Klaes et al. 2001; Bibbo et al. 2002). In all normal cervical tissues examined, no p16^INK4A^ staining is evident. Additionally, all normal regions adjacent to cervical intraepithelial neoplasia (CIN) lesions do not show any detectable expression of p16^INK4A^. While p16^INK4A^ identified dysplastic squamous and glandular lesions with a sensitivity rate of 99.9% and a specificity rate of 100% in cervical biopsy sections, only a few studies have examined the possible prognostic value of p16^INK4A^ in cervical lesions (Murphy et al. 2003). It is now widely accepted that p16^INK4A^ is a sensitive and specific marker of squamous and glandular dysplastic cells of the cervix and also a surrogate marker of high risk human papillomavirus, suggesting a valuable adjunctive test in cervical cancer screening (Table 4).

### Squamous cell carcinoma antigen (SCC)

SCC belongs to the family of serine and cysteine protease inhibitors (Suminami et al. 1991). This antigen is present in normal cervix epithelium with an increased expression in proportion to dyspalstic lesion and cervical squamous cell carcinoma. Though SCC is not sufficient for use in screening, pretreatment serum SCC values works as an independent prognostic factor. Approximately 60% of patients with cervical cancer are detected with elevated levels of serum SCC at initial diagnosis, when all stages are included (Farghaly, 1992). Besides, serum SSC -> SCC levels correlate significantly with tumor stage (Crombach et al. 1989; Duk et al. 1990). More specifically -> If split with stage, serum SCC is elevated in 24–53% of patients with Stage IB or IIA squamous cell cervical cancer, and in 75–90% of patients with advanced stage (FIGO IIB and higher) disease (Gaarenstroom et al. 1995; Duk et al. 1996). Several studies have concluded that serum SCC is useful in monitoring the course of squamous cell cervical cancer following primary therapy (Bolli et al. 1994; Bonfrer et al. 1997). Persistently elevated and/or increasing serum SCC levels after and/or during treatment suggest tumor persistence or progressive disease (Brioschi et al. 1991). Patients with plateau SCC level revealed higher incidence of treatment failure after radiotherapy, indicating SCC levels provide useful information for the need of further work-up and management (Hong et al. 1998). In view of a strong correlation with the clinical course, SCC is suitable for monitoring the early detection of recurrent or progressive disease after primary treatment, and may therefore be useful in the management of patients. However, there is as yet no evidence that earlier detection of recurrent disease influences treatment outcome (Table 5).

### Cell proliferation markers

The rate of cell proliferation in a tumor is generally thought to be of prognostic importance, and until recently the only means available to the pathologist to assess this was to count the number of mitotic figures, a technique fraught with difficulties and pitfalls. A number of antigens have now been described, which are expressed specifically by proliferating cells and which, with the use of monoclonal antibodies, can be demonstrated immunocytochemically: demonstration of these antigens affords, in theory at least, a much more accurate estimate of the number of proliferating cells than does a mitotic count. The two proliferation antigens which have been most widely studied are proliferating cell nuclear antigen (PCNA), which is expressed during the G1 and early S phases of the proliferative cycle, and Ki-67, which is expressed during the G2 and mitotic phases of the cycle. Ki-67 is the more reliable indicator of the growth fraction of a tumor, largely because PCNA has a long half-life and may still be demonstrable in post-mitotic cells (Scott et al. 1991). The study of Ki-67 was originally, however, limited by the necessity to use fresh or snap frozen tissue (Hall and Levison, 1990), but the recently introduced antibody MIB-1 can be used to detect the antigen in fixed paraffin-embedded tissue (McCormick et al. 1993). The number of cell nuclei staining positively for these markers of proliferation can be estimated by simple counting or can be measured in an image analysis system. In cervical intraepithelial neoplasia both PCNA and Ki-67 expressions are, as compared to normal cervices, increased in the upper levels of the cervical epithelium (Konishi et al. 1991; Shurbaji et al. 1993; Mittal et al. 1993; Raju, 1994; McLuggage et al. 1996), and it is thought that this staining pattern, particularly that for Ki-67, may be of considerable value in distinguishing CIN from non-neoplastic lesions that may mimic CIN. Two studies of PCNA expression in cervical carcinoma have yielded conflicting results, one finding the PCNA index to be of considerable import (Oka et al. 1992) and another being unable to show that this index is of any prognostic value (Al-Nafussi et al. 1993). Investigations of Ki-67 expression in cervical carcinoma have generally failed to show any relationship between the number of positively stained cells and prognosis (Cole et al. 1992; Levine et al. 1995; Oka and Arai, 1996), though in one study the Ki-67 index was significantly related to tumor size, lymphatic spread, and disease-free interval in patients with stage I disease (Garzetti et al. 1995). In endometrial adenocarcinomas the PCNA index has been found to correlate with tumor grade, depth of myometrial invasion, and recurrence risk (Garzetti et al. 1996a), and it has been suggested that PCNA staining can be used as a method of pre-operative identification of high risk patients (Garzetti et al. 1996b). Ki-67expression in endometrial carcinomas was found to be correlated with grade but not with stage or depth of myometrial invasion in one study (Nielsen et al. 1994), but it emerged as a highly significant indicator of tumor recurrence in another (Geislet et al. 1996) (Table 6). By contrast, others have found neither staining for PCNA nor Ki-67 to be of any prognostic value in endometrial neoplasm (Hamel et al. 1996; Nordstrom et al. 1996).

Although few new markers have reached the clinic in recent years, several reported cancer biomarkers have been found to have low sensitivity in that they are found only in a small subset of patients with a particular type of cancer.

## Needs of Biomarker Discovery

The future of clinical cancer management belongs to the prognostic and predictive biomarkers of cancer. These markers are of utmost importance as they will be the used to make clinical decisions that will eventually save lives. In the future, biomarkers will guide decision making during cancer management. Biomarkers that correctly predict outcome in a specific disease and allow physicians and patients to make informed treatment decisions need to be developed. Biomarkers will not only help screen, detect, diagnose, help in prognostic evaluation, monitor treatment and predict recurrence, but also play a major role in clinical decision making.

## New Biomarker Development

Concern remains as to whether the tools available are well suited to provide the technological support to meet the demands of new biomarker development. Until recently, the discovery of cancer biomarkers has been a slow approach to identify proteins that are dysregulated as a consequence of the disease and shed into the body fluids such as serum, urine or saliva. Unfortunately, this approach is arduous and prolonged as each candidate markers must be identified among thousands of proteins. The recent advancements in genomic and proteomic technologies including gene array technology, serial analysis of gene expression (SAGE) improved 2-DE and new mass spectrometric techniques coupled with advancements in bioinformatic tools, shows great promise of meeting the demand for the discovery of a variety of new biomarkers that are both sensitive and specific (Chatterjee and Zetter, 2005). Like these, high-throughput approaches are useful in cancer biomarker discovery and clinical diagnostics. The combined use of proteomics, genomics and bioinformatics tools may hold promise for early detection of disease by proteomic patterns, diagnosis based on proteomic signatures as a complement to histopathology, individualized selection of therapeutic combinations that best target the entire disease-specific protein network, rational modulation of therapy based on changes in the diseased protein network associated with drug resistance and understanding of carcinogenesis.

## Figures and Tables

**Table 1:** The single Pap smear test has limited sensitivity and specificity.

Limitations of Pap smear screening
For a high grade lesion, the sensitivity of a single pap smear is only 60–80% Errors in sampling, slide preparation and interpretation are inherent in cytology Sampling for atypical glandular cells is exceptionally difficult False-positive rates range from 15–50% False-negative rates may reach 30%

**Table 2:** MCM5 and HPV oncoprotein expression.

MCM5
MCM5 overexpression may be due to the release of Rb inhibition on transcription factor E2F due to binding of HPV E7 oncoproteins E2F may bind to the MCM5 promoter to increase transcription of MCM5 MCM5 mRNA expression increase significantly with increasing severity of dysplasia

**Table 3:** CDC6 and HPV oncoprotein expression.

CDC6
Inactivation of Rb by HPV E7 - Release inhibitor of E2F - May transcriptionally up-regulate CDC6 CDC6 mRNA expression is significantly increased in high-grade dysplastic cells Overexpressionof CDC6 promotes re-replication, genomic instability and DNA damage in human cancer cells with inactive p53, but not in cells with functional p53 High-risk HPV E6 oncoprotein targets p53 for proteolytic degradation, allowing re-replication to occur in the presence of CDC6 overpression

**Table 4:** p16^INK4A^ and HPV oncoprotein expression.

p16^INK4A^
Inactivation of Rb by HPV E7 protein may up-regulate p16^INK4A^ p16^INK4A^ may be directly induced by the transcription factor E2F released from pRb after binding of HPV E7 An HPV-independent pathway for p16^INK4A^ up-regulation many also exist

**Table 5:** Currently available and potentially useful serum marker squamous cell carcinoma (SCC).

Squamous cell carcinoma (SCC)
Pre-treatment identification of high risk group with lymph node metastases in quamous cell cervical cancer Pre-treatment prediction of prognosis in squamous cell cervical cancer Prediction of response to treatment in squamous cell cervical cancer Monitoring disease and detecting recurrent disease in squamous cell cervical cancer

**Table 6:** Cell proliferation markers PCNA and Ki-67.

	Type	Limitation
PCNA	Proliferation marker	Multiple factors affect staining intensity
Ki-67	Proliferation marker	Multiple factors affect expression levels

## References

[b1-bmi-2006-215] Alison MR Hunt T Forbes SJ 2002 Minichromosome maintenance (MCM) proteins may be pre-cancer markers Gut 50 290 1 11839701 10.1136/gut.50.3.290 PMC1773123

[b2-bmi-2006-215] Al-Nafussi AI Klys HS Rebello G 1993 The assessment of proliferating cell nuclear antigen (PCNA) immunostaining in the uterine cervix and cervical squamous neoplasia Int J Gynecol Cancer 3 154 8 11578336 10.1046/j.1525-1438.1993.03030154.x

[b3-bmi-2006-215] Antinore MJ Birrer MJ Patel D 1996 The human papillomavirus type 16 E7 gene product interacts with and trans-activates the AP1 family of transcription factors EMBO J 15 1950 60 8617242 PMC450114

[b4-bmi-2006-215] Antonsson A Payne E Hengst K 2006 The Human Papillomavirus Type 16 E7 Protein Binds Human Interferon Regulatory Factor-9 via a Novel PEST Domain Required for Transformation J Interferon Cytokine Res 26 455 61 16800784 10.1089/jir.2006.26.455

[b5-bmi-2006-215] Autier P Coibion M De Sutter P 1999 Cytology alone versus cytology and cervicography for cervical cancer screening: a randomized study Obstet Gynecol 93 353 8 10074978 10.1016/s0029-7844(98)00472-4

[b6-bmi-2006-215] Barnard P McMillan NA 1999 The human papillomavirus E7 oncoprotein abrogates signaling mediated by interferon-alpha Virology 259 305 13 10388655 10.1006/viro.1999.9771

[b7-bmi-2006-215] Bemard B Pretet JL Charlot JF 2003 Human papillomaviruses type 16+ and 18+ cervical carcinoma cells are sensitive to staurosporine-mediated apoptosis Biol Cell 95 17 26 12753950 10.1016/s0248-4900(02)01220-0

[b8-bmi-2006-215] Bibbo M Klump WJ DeCecco J 2002 Procedure for immunocytochemical detection of P16INK4 antigen in thin-layer, liquid-based specimens Acta Cytologica 46 25 9 11843554 10.1159/000326711

[b9-bmi-2006-215] Bolli JN Doering DL Bosscher JR 1994 Squamous cell carcinoma antigen: clinical utility in squamous cell carcinoma of the uterine cervix Gynecol Oncol 55 169 73 7959279 10.1006/gyno.1994.1272

[b10-bmi-2006-215] Brehm A Nielsen SJ Miska EA 1999 The E7 oncoprotein associates with Mi2 and histone deacetylase activity to promote cell growth EMBO J 18 2449 58 10228159 10.1093/emboj/18.9.2449 PMC1171327

[b11-bmi-2006-215] Brioschi PA Bischof P Delafosse C 1991 Squamous cell carcinoma antigen (SCC-A) values related to clinical outcome of pre-invasive and invasive cervical carcinoma Int J Cancer 47 376 9 1704354 10.1002/ijc.2910470311

[b12-bmi-2006-215] Brotzman GL Spitzer M Apgar BS Brotzman GL Spitzer M 2002 Adjunctive testing: cervicography Colposcopy: Principles and practice Philadelphia Saunders 73 84

[b13-bmi-2006-215] Chatterjee SK Zetter BR 2005 Cancer biomarkers: knowing the present and predicting the future Future Oncol 1 37 50 16555974 10.1517/14796694.1.1.37

[b14-bmi-2006-215] Chen JJ Reid CE Band V 1995 Interaction of papillomavirus E6 oncoproteins with a putative calcium-binding protein Science 269 529 31 7624774 10.1126/science.7624774

[b15-bmi-2006-215] Chen TM Defendi V 1992 Functional interaction of p53 with HPV18 E6, c-myc and H-ras in 3T3 cells Oncogene 7 1541 7 1321402

[b16-bmi-2006-215] Cole DJ Brown DC Crossley F 1992 Carcinoma of the cervix uteri: An assessment of the relationship of tumor proliferation to prognosis Br J Cancer 65 783 5 1586610 10.1038/bjc.1992.167 PMC1977376

[b17-bmi-2006-215] Cook JG Park CH Burke TW 2002 Analysis of Cdc6 function in the assembly of mammalian prereplication complexes Proc Natl Acad Sci USA 99 1347 52 11805305 10.1073/pnas.032677499 PMC122193

[b18-bmi-2006-215] Costa S Sideri M Syrjanen K 2000 Combined Pap smear, cervicography and HPV DNA testing in the detection of cervical intraepithelial neoplasia and cancer Acta Cytol 44 310 8 10833984 10.1159/000328471

[b19-bmi-2006-215] Crombach G Würz H Herrmann F 1989 Bedeutung des SCC-antigens in der diagnostik und verlaufskontrolle des zervixkarzinoms Dtsch Med Wochenschr 114 700 5 2714197 10.1055/s-2008-1066658

[b20-bmi-2006-215] Davidson EJ Morris LS Scott IS 2003 Minichromosome maintenance (Mcm) proteins, cyclin B1 and D1, phosphohistone H3 and in situ DNA replication for functional analysis of vulval intraepithelial neoplasia Br J Cancer 88 257 62 12610511 10.1038/sj.bjc.6600729 PMC2377046

[b21-bmi-2006-215] Davies RJ Freeman A Morris LS 2002 Analysis of minichromosome maintenance proteins as a novel method for detection of colorectal cancer in stool Lancet 369 1917 9 12057556 10.1016/S0140-6736(02)08739-1

[b22-bmi-2006-215] Davies R Hicks R Crook T 1993 Human papillomavirus type 16 E7 associates with a histone H1 kinase and with p107 through sequences necessary for transformation J Virol 67 2521 8 8386265 10.1128/jvi.67.5.2521-2528.1993 PMC237571

[b23-bmi-2006-215] Degenhardt YY Silverstein SJ 2001 Gps2, a protein partner for human papillomavirus E6 proteins J Virol 75 151 60 11119584 10.1128/JVI.75.1.151-160.2001 PMC113908

[b24-bmi-2006-215] Degenhardt YY Silverstein SJ 2001 Interaction of zyxin, a focal adhesion protein, with the e6 protein from human papillomavirus type 6 results in its nuclear translocation J Virol 75 11791 802 11689660 10.1128/JVI.75.23.11791-11802.2001 PMC114765

[b25-bmi-2006-215] DeMay RM 1996 Cytopathology of false negativespreceding cervical carcinoma Am J Obstet Gynecol 175 1110 3 8885795 10.1016/s0002-9378(96)70013-3

[b26-bmi-2006-215] Doorbar J 2006 Molecular biology of human papillomavirus infection and cervical cancer Clin Sci (Lond) 110 525 41 16597322 10.1042/CS20050369

[b27-bmi-2006-215] Du M Fan X Hong E 2002 Interaction of oncogenic papillomavirus E6 proteins with fibulin-1 Biochem Biophys Res Commun 296 962 9 12200142 10.1016/s0006-291x(02)02041-7

[b28-bmi-2006-215] Duensing S Munger K 2003 Human papillomavirus type 16 E7 oncoprotein can induce abnormal centrosome duplication through a mechanism independent of inactivation of retinoblastoma protein family members J Virol 77 12331 5 14581569 10.1128/JVI.77.22.12331-12335.2003 PMC254291

[b29-bmi-2006-215] Duk JM De Bruijn HWA Groenier KH 1990 Cancer of the uterine cervix: sensitivity and specificity of serum squamous cell carcinoma antigen determinations Gynecol Oncol 39 186 94 2227594 10.1016/0090-8258(90)90430-s

[b30-bmi-2006-215] Duk JM Groenier KH De Bruijn HWA 1996 Pretreatment serum squamous cell carcinoma antigen: a newly identified prognostic factor in early-stage cervical carcinoma J Clin Oncol 14 111 8 8558185 10.1200/JCO.1996.14.1.111

[b31-bmi-2006-215] Dyson N Guida P Münger K 1992 Homologous sequences in adenovirus E1A and human papillomavirus E7 proteins mediate interaction with the same set of cellular proteins J Virol 66 6893 902 1331501 10.1128/jvi.66.12.6893-6902.1992 PMC240306

[b32-bmi-2006-215] Dyson N Howley PM Münger K 1989 The human papillomavirus-16 E7 oncoprotein is able to bind to the retinoblastoma gene product Science 243 934 40 2537532 10.1126/science.2537532

[b33-bmi-2006-215] Farghaly SA 1992 Tumor markers in gynecologic cancer Gynecol Obstet Invest 34 65 72 1398266 10.1159/000292728

[b34-bmi-2006-215] Felix JC 2003 The science behind the effectiveness of in vivo screening Am J Obstet Gynecol 188 8 12 10.1067/mob.2003.235 12634625

[b35-bmi-2006-215] Felix JC Lonky NM Tamura K 2002 Aberrant expression of E-cadherin in cervical intraepithelial neoplasia correlates with a falsenegative Papanicolaou smear Am J Obstet Gynecol 186 1308 14 12066114 10.1067/mob.2002.123732

[b36-bmi-2006-215] Ferris DG Schiffman M Litaker MS 2001 Cervicography for triage of women with mildly abnormal cervical cytology results Am J Obstet Gynecol 185 939 43 11641682 10.1067/mob.2001.117485

[b37-bmi-2006-215] Filippova M Parkhurst L Duerksen-Hughes PJ 2004 The human papillomavirus 16 E6 protein binds to Fas-associated death domain and protects cells from Fas-triggered apoptosis J Biol Chem 279 25729 44 15073179 10.1074/jbc.M401172200

[b38-bmi-2006-215] Filippova M Song H Connolly JL 2002 The human papillomavirus 16 E6 protein binds to tumor necrosis factor (TNF) R1 and protects cells from TNF-induced apoptosis J Biol Chem 277 21730 9 11934887 10.1074/jbc.M200113200

[b39-bmi-2006-215] Freeman A Morris LS Mills AD 1999 Minichromosome maintenance proteins as biological markers of dysplasia and malignancy Clin Cancer Res 5 121 32 10473096

[b40-bmi-2006-215] Gaarenstroom KN Bonfrer JMG Kenter GG 1995 Clinical value of pre-treatment serum Cyfra 21–1, tissue polypeptide antigen, and squamous cell carcinoma antigen levels in patients with cervical cancer Cancer 76 807 13 8625184 10.1002/1097-0142(19950901)76:5<807::aid-cncr2820760515>3.0.co;2-m

[b41-bmi-2006-215] Gao Q Kumar A Srinivasan S 2000 PKN binds and phosphorylates human papillomavirus E6 oncoprotein J Biol Chem 275 14824 30 10809724 10.1074/jbc.275.20.14824

[b42-bmi-2006-215] Gao Q Srinivasan S Boyer SN 1999 The E6 oncoproteins of high-risk papillomaviruses bind to a novel putative GAP protein, E6TP1, and target it for degradation Mol Cell Biol 19 733 44 9858596 10.1128/mcb.19.1.733 PMC83930

[b43-bmi-2006-215] Garzetti GG Ciavittini A Goteri G 1996a Proliferating cell nuclear antigen (PCNA) immunoreactivity in stage I endometrial cancer: A new prognostic factor Int J Gynecol Cancer 6 186 92

[b44-bmi-2006-215] Garzetti GG Ciavittini A Goteri G 1996b Proliferating cell nuclear antigen in endometrial adenocarcinoma: Pre-treatment identification of high risk patients Gynecol Oncol 61 16 21 8626110 10.1006/gyno.1996.0089

[b45-bmi-2006-215] Garzetti GG Ciavittini A Lucarini G 1995 MIB1 immunostaining in stage 1 squamous cervical carcinoma: relationship with natural killer cell activity Gynecol Oncol 58 23 33 10.1006/gyno.1995.1179 7789886

[b46-bmi-2006-215] Geisler JP Wiemann MC Zhou Z 1996 Proliferation index determined by MIB-1 and recurrence of endometrial carcinoma Gynecol Oncol 61 373 7 8641618 10.1006/gyno.1996.0159

[b47-bmi-2006-215] Glaunsinger BA Lee SS Thomas M 2000 Interactions of the PDZ-protein MAGI-1 with adenovirus E4-ORF1 and high-risk papillomavirus E6 oncoproteins Oncogene 19 5270 80 11077444 10.1038/sj.onc.1203906 PMC3072458

[b48-bmi-2006-215] Going JJ Keith WN Neilson L 2002 Aberrant expression of minichromosome maintenance proteins 2 and 5, and Ki-67 in dysplastic squamous oesophageal epithelium and Barrett’s mucosa Gut 50 373 7 11839717 10.1136/gut.50.3.373 PMC1773132

[b49-bmi-2006-215] Gygi SP Aebersold R 2000 Mass spectrometry and proteomics Curr Opin Chem Biol 4 489 94 11006534 10.1016/s1367-5931(00)00121-6

[b50-bmi-2006-215] Habig M Smola H Dole VS 2006 E7 proteins from high- and low-risk human papillomaviruses bind to TGF-beta-regulated Smad proteins and inhibit their transcriptional activity Arch Virol 19 [Epub ahead of print] 10.1007/s00705-006-0768-1 16710631

[b51-bmi-2006-215] Hall PA LEvison DA 1990 Assessment of cellular proliferation in histological material J Clin Pathol 43 184 92 2185282 10.1136/jcp.43.3.184 PMC502326

[b52-bmi-2006-215] Hamel NW Sebo TJ Wilson TO 1996 Prognostic value of p53 and proliferating cell nuclear antigen expression in endometrial carcinoma Gynecol Oncol 62 192 8 8751548 10.1006/gyno.1996.0214

[b53-bmi-2006-215] Hong JH Tsai CS Chang JT 1998 The prognostic significance of pre-and post-treatment SCC levels in patients with squamous cell carcinoma of the cervix treated by radiotherapy Int J Radiat Oncol Biol Phys 41 823 30 9652844 10.1016/s0360-3016(98)00147-3

[b54-bmi-2006-215] Huang SM McCance DJ 2002 Down regulation of the interleukin-8 promoter by human papillomavirus type 16 E6 and E7 through effects on CREB binding protein/p300 and P/CAF J Virol 76 8710 21 12163591 10.1128/JVI.76.17.8710-8721.2002 PMC136974

[b55-bmi-2006-215] Iftner T Elbel M Schopp B 2002 Interference of papillomavirus E6 protein with single-strand break repair by interaction with XRCC1 EMBO J 21 4741 8 12198176 10.1093/emboj/cdf443 PMC126183

[b56-bmi-2006-215] Jones DL Alani RM Munger K 1997 The human papillomavirus E7 oncoprotein can uncouple cellular differentiation and proliferation in human keratinocytes by abrogating p21Cip1-mediated inhibition of cdk2 Genes Dev 11 2101 11 9284049 10.1101/gad.11.16.2101 PMC316455

[b57-bmi-2006-215] Kiyono T Hiraiwa A Fujita M 1997 Binding of high-risk human papillomavirus E6 oncoproteins to the human homologue of the Drosophila discs large tumor suppressor protein Proc Natl Acad Sci USA 94 11612 6 9326658 10.1073/pnas.94.21.11612 PMC23554

[b58-bmi-2006-215] Klaes R Friedrich T Spitkovsky D 2001 Overexpression of p16(INK4A) as a specific marker for dysplastic and neoplastic epithelial cells of the cervix uteri Int J Cancer 92 276 84 11291057 10.1002/ijc.1174

[b59-bmi-2006-215] Konishi I Jujii S Nonogaki H 1991 Immunohistochemical analysis of estrogen receptors, progesterone receptors, Ki-67 antigen, and human papillomavirus DNA in normal and neoplastic epithelium of the uterine cervix Cancer 68 1340 50 1651807 10.1002/1097-0142(19910915)68:6<1340::aid-cncr2820680626>3.0.co;2-q

[b60-bmi-2006-215] Kukimoto I Aihara S Yoshiike K 1998 Human papillomavirus oncoprotein E6 binds to the C-terminal region of human minichromosome maintenance 7 protein Biochem Biophys Res Commun 249 258 62 9705868 10.1006/bbrc.1998.9066

[b61-bmi-2006-215] Kumar A Zhao Y Meng G 2002 Human papillomavirus oncoprotein E6 inactivates the transcriptional coactivator human ADA3 Mol Cell Biol 22 5801 12 12138191 10.1128/MCB.22.16.5801-5812.2002 PMC133989

[b62-bmi-2006-215] Lei M Tye BK 2001 Initiating DNA synthesis: from recruiting to activating the MCM complex J Cell Sci 114 1447 54 11282021 10.1242/jcs.114.8.1447

[b63-bmi-2006-215] Lee SS Glaunsinger B Mantovani F 2000 Multi-PDZ domain protein MUPP1 is a cellular target for both adenovirus E4-ORF1 and high-risk papillomavirus type 18 E6 oncoproteins J Virol 74 9680 93 11000240 10.1128/jvi.74.20.9680-9693.2000 PMC112400

[b64-bmi-2006-215] Levine EL Renehan A Gossiel R 1995 Apoptosis, intrinsic radiosensitivity and prediction of radiotherapy response in cervical carcinoma Radiother Oncol 37 1 9 8539450 10.1016/0167-8140(95)01622-n

[b65-bmi-2006-215] Lonky NM Edwards G 1992 Chemiluminescent light versus incandescent light in the visualization of acetowhite epithelium Am J Gynecol Health 6 11 5

[b66-bmi-2006-215] Lonky NM Apgar BS Brotzman GL Spitzer M 2002 Adjunctive testing: cervical screening with *in vivo* and *in vitro* modalities: speculoscopy combined with cytology Colposcopy: Principles and practice Philadelphia Saunders 98 106

[b67-bmi-2006-215] Luscher-Firzlaff JM Westendorf JM Zwicker J 1999 Interaction of the fork head domain transcription factor MPP2 with the human papilloma virus 16 E7 protein: enhancement of transformation and transactivation Oncogene 18 5620 30 10523841 10.1038/sj.onc.1202967

[b68-bmi-2006-215] Mann W Lonky N Massad S 1993 Papanicolaou smear screening augmented by a magnified chemiluminescent exam Int J Gynecol Obstet 43 289 96 10.1016/0020-7292(93)90518-2 7907040

[b69-bmi-2006-215] Mannhardt B Weinzimer SA Wagner M 2000 Human papillomavirus type 16 E7 oncoprotein binds and inactivates growth-inhibitory insulin-like growth factor binding protein 3 Mol Cell Biol 20 6483 95 10938125 10.1128/mcb.20.17.6483-6495.2000 PMC86123

[b70-bmi-2006-215] Mazzarelli JM Atkins GB Geisberg JV 1995 The viral oncoproteins Ad5 E1A, HPV16 E7 and SV40 TAg bind a common region of the TBP-associated factor-110 Oncogene 11 1859 64 7478615

[b71-bmi-2006-215] McCormick D Chong H Hobbs C 1993 Detection of the Ki67 antigen in fized and wax embedded sections with the monoclonal antibody MIB1 Histopathology 22 355 60 8514278 10.1111/j.1365-2559.1993.tb00135.x

[b72-bmi-2006-215] McGoogan E Colgan TJ Ramzy I 1998 Cell preparation methods and criteria for sample adequacy. IAC Task Force summary. Diagnostic cytology towards the 21st century: An international expert conference and tutorial Acta Cytol 42 25 32 9479321 10.1159/000331532

[b73-bmi-2006-215] McIntyre MC Ruesch MN Laimins LA 1996 Human papillomavirus E7 oncoproteins bind a single form of cyclin E in a complex with cdk2 and p107 Virology 215 73 82 8553588 10.1006/viro.1996.0008

[b74-bmi-2006-215] McLuggage WG Buhidma M Tang L 1996 Monoclonal antibody MIB1 in the assessment of cervical squamous intraepithelial lesions Int J Gynecol Pathol 15 131 6 8786202 10.1097/00004347-199604000-00007

[b75-bmi-2006-215] Mittal KR Demopoulos RJ Goswami S 1993 Proliferating cell nuclear antigen (cyclin) expression in normal and abnormal squamous epithelia Am J surg Pathol 17 117 22 8422109 10.1097/00000478-199302000-00003

[b76-bmi-2006-215] Murphy N Ring M Killalea AG 2003 p16INK4 as a marker for cervical dyskaryosis: CIN and cGIN in cervical biopsies and ThinPrep smears J Clin Pathol 56 56 63 12499437 10.1136/jcp.56.1.56 PMC1769860

[b77-bmi-2006-215] Nakagawa S Huibregtse JM 2000 Human scribble (Vartul) is targeted for ubiquitin-mediated degradation by the high-risk papillomavirus E6 proteins and the E6AP ubiquitin-protein ligase Mol Cell Biol 20 8244 53 11027293 10.1128/mcb.20.21.8244-8253.2000 PMC86433

[b78-bmi-2006-215] Negm RS Verma M Srivastava S 2002 The promise of biomarkers in cancer screening and detection Trends Mol Med 8 288 93 12067615 10.1016/s1471-4914(02)02353-5

[b79-bmi-2006-215] Nielsen AL Nyholm HCJ Engel P 1994 Expressionof MIB-1 (paraffin ki-67) and AgNOR morphology in endometrial adenocarcinomas of endometrioid type Int J Gynecol Pathol 13 37 44 8112955 10.1097/00004347-199401000-00005

[b80-bmi-2006-215] Nordstrom B Strang P Bergstrom R 1996 A comparison of proliferation markers and their prognostic value for woman with endometrial carcinoma: Ki-67, proliferating cell nuclear antigen and flow cytometric S-phase factor Cancer 78 1942 51 8909315

[b81-bmi-2006-215] Ohta S Koide M Tokuyama T 2001 Cdc6 expression as a marker of proliferative activity in brain tumors Oncol Rep 8 1063 6 11496317 10.3892/or.8.5.1063

[b82-bmi-2006-215] Oka K Arai T 1996 MIB1 growth fraction is not related to prognosis in cervical squamous cell carcinoma treated with radiotherapy Int J Gynecol Pathol 15 23 7 8852442 10.1097/00004347-199601000-00004

[b83-bmi-2006-215] Oka K Hoshi T Arai T 1992 Prognostic significance of the PC10 index as a prospective assay for cervical cancer treated with radiation therapy alone Cancer 70 1545 50 1355399 10.1002/1097-0142(19920915)70:6<1545::aid-cncr2820700617>3.0.co;2-s

[b84-bmi-2006-215] Parham GP 2003 Comparison of cell collection and direct visualization cervical cancer screening adjuncts Am J Obstet Gynecol 188 S13 20 12634626 10.1067/mob.2003.234

[b85-bmi-2006-215] Park JS Kim EJ Kwon HJ 2000 Inactivation of interferon regulatory factor-1 tumor suppressor protein by HPV E7 oncoprotein. Implication for the E7-mediated immune evasion mechanism in cervical carcinogenesis J Biol Chem 275 6764 9 10702232 10.1074/jbc.275.10.6764

[b86-bmi-2006-215] Raju GC 1994 Expression of the proliferating cell nuclear antigen in cervical neoplasia Int J Gynecol Pathol 13 337 41 7814195 10.1097/00004347-199410000-00007

[b87-bmi-2006-215] Ronco LV Karpova AY Vidal M 1998 Human papillomavirus 16 E6 oncoprotein binds to interferon regulatory factor-3 and inhibits its transcriptional activity Genes Dev 12 2061 72 9649509 10.1101/gad.12.13.2061 PMC316980

[b88-bmi-2006-215] Sano T Oyama T Kashiwabara K 1998 Immunohistochemical overexpression of p16 protein associated with intact retinoblastoma protein expression in cervical cancer and cervical intraepithelial neoplasia Pathol Int 48 580 5 9736404 10.1111/j.1440-1827.1998.tb03954.x

[b89-bmi-2006-215] Schilling B De-Medina T Syken J 1998 A novel human DnaJ protein, hTid-1, a homolog of the Drosophila tumor suppressor protein Tid56, can interact with the human papillomavirus type 16 E7 oncoprotein Virology 247 74 85 9683573 10.1006/viro.1998.9220

[b90-bmi-2006-215] Schneider DL Herrero R Bratti C 1999 Cervicography screening for cervical cancer among 8,460 women in a high-risk population Am J Obstet Gynecol 180 290 8 9988789 10.1016/s0002-9378(99)70202-4

[b91-bmi-2006-215] Scott RJ Hall PA Haldane JS 1991 A comparison of immunohistochemical markers of cell proliferation with experimentally determined growth fraction J Pathol 165 173 8 1683905 10.1002/path.1711650213

[b92-bmi-2006-215] Shin JH Grabowski B Kasiviswanathan R 2003 Regulation of minichromosome maintenance helicase activity by Cdc6 J Biol Chem 278 38059 67 12837750 10.1074/jbc.M305477200

[b93-bmi-2006-215] Shurbaji MS Brooks SK Thurmond TS 1993 Proliferating cell nuclear antigen: Immunoreactivity in cervical intraepithelial neoplasia and benign cervical epithelium Am J Clin Pathol 100 22 6 8102221 10.1093/ajcp/100.1.22

[b94-bmi-2006-215] Spitzer M 2002 In vitro conventional cytology: Historical strengths and current limitations Obstet Gynecol Clin North Am 29 673 83 12509091 10.1016/s0889-8545(02)00025-6

[b95-bmi-2006-215] Srivenugopal KS Ali-Osman F 2002 The DNA repair protein, O(6)-methylguanine-DNA methyltransferase is a proteolytic target for the E6 human papillomavirus oncoprotein Oncogene 21 5940 5 12185595 10.1038/sj.onc.1205762

[b96-bmi-2006-215] Stoeber K Swinn R Prevost AT 2002 Diagnosis of genitor-urinary tract cancer by detection of minichromosome maintenance 5 protein in urine sediments J Natl Cancer Inst 94 1071 9 12122098 10.1093/jnci/94.14.1071

[b97-bmi-2006-215] Suminami Y Kishi F Sekiguchi K 1991 Squamous cell carcinoma antigen is a new member of the serine protease inhibitors Biochem Biophys Res Commun 181 51 8 1958219 10.1016/s0006-291x(05)81380-4

[b98-bmi-2006-215] Thomas M Banks L 1999 Human papillomavirus (HPV) E6 interactions with Bak are conserved amongst E6 proteins from high and low risk HPV types J Gen Virol 80 1513 7 10374970 10.1099/0022-1317-80-6-1513

[b99-bmi-2006-215] Thomas M Laura R Hepner K 2002 Oncogenic human papillomavirus E6 proteins target the MAGI-2 and MAGI-3 proteins for degradation Oncogene 21 5088 96 12140759 10.1038/sj.onc.1205668

[b100-bmi-2006-215] Thomas M Pim D Banks L 1999 The role of the E6–p53 interaction in the molecular pathogenesis of HPV Oncogene 18 7690 700 10618709 10.1038/sj.onc.1202953

[b101-bmi-2006-215] Tong X Howley PM 1997 The bovine papillomavirus E6 oncoprotein interacts with paxillin and disrupts the actin cytoskeleton Proc Natl Acad Sci USA 94 4412 7 9114003 10.1073/pnas.94.9.4412 PMC20736

[b102-bmi-2006-215] Walboomers JM Jacobs MV Manos MM 1999 Human papillomavirus is a necessary cause of invasive cervical cancer worldwide J Pathol 189 12 9 10451482 10.1002/(SICI)1096-9896(199909)189:1<12::AID-PATH431>3.0.CO;2-F

[b103-bmi-2006-215] Wilkins MR Sanchez JC Gooley JC 1995 Progress with proteome projects: why all proteins expressed by a genome should be identified and how to do it Biotechnol Gene Eng Rev 13 19 50 10.1080/02648725.1996.10647923 8948108

[b104-bmi-2006-215] Williams GH Romanowski P Morris L 1998 Improved cervical smear assessment using antibodies against proteins that regulate DNA replication Proc Natl Acad Sci USA 95 14932 7 9843993 10.1073/pnas.95.25.14932 PMC24553

[b105-bmi-2006-215] Wright TC Jr Denny L Kuhn L 2002 Use of visual screening methods for cervical cancer screening Obstet Gynecol Clin N Am 29 701 34 10.1016/s0889-8545(02)00045-1 12509093

[b106-bmi-2006-215] Yim EK Meoyng J Namakoong SE 2004 Genomic and proteomic expression patterns in HPV-16 E6 gene transfected stable human carcinoma cell lines DNA Cell Biol 23 826 35 15684709 10.1089/dna.2004.23.826

[b107-bmi-2006-215] Zerfass-Thome K Zwerschke W Mannhardt B 1996 Inactivation of the cdk inhibitor p27KIP1 by the human papillomavirus type 16 E7 oncoprotein Oncogene 13 2323 30 8957073

[b108-bmi-2006-215] Zwerschke W Mannhardt B Massimi P 2000 Allosteric activation of acid alpha-glucosidase by the human papillomavirus E7 protein J Biol Chem 275 9534 41 10734102 10.1074/jbc.275.13.9534

[b109-bmi-2006-215] Zwerschke W Mazurek S Massimi P 1999 Modulation of type M2 pyruvate kinase activity by the human papillomavirus type 16 E7 oncoprotein Proc Natl Acad Sci USA 96 1291 6 9990017 10.1073/pnas.96.4.1291 PMC15456

